# The floral transcriptomes of four bamboo species (Bambusoideae; Poaceae): support for common ancestry among woody bamboos

**DOI:** 10.1186/s12864-016-2707-1

**Published:** 2016-05-20

**Authors:** William P. Wysocki, Eduardo Ruiz-Sanchez, Yanbin Yin, Melvin R. Duvall

**Affiliations:** Biological Sciences, Northern Illinois University, 1425 W Lincoln Hwy, DeKalb, IL 60115-2861 USA; Escuela Nacional de Estudios Superiores Unidad Morelia (UNAM), Antigua Carretera a Pátzcuaro 8701, Col. Ex Hacienda de San José de la Huerta, Morelia, Michoacán 58190 Mexico

**Keywords:** Transcriptome, Bambusoideae, Woody bamboos, RNA-Seq, MADS-box

## Abstract

**Background:**

Next-generation sequencing now allows for total RNA extracts to be sequenced in non-model organisms such as bamboos, an economically and ecologically important group of grasses. Bamboos are divided into three lineages, two of which are woody perennials with bisexual flowers, which undergo gregarious monocarpy. The third lineage, which are herbaceous perennials, possesses unisexual flowers that undergo annual flowering events.

**Results:**

Transcriptomes were assembled using both reference-based and de novo methods. These two methods were tested by characterizing transcriptome content using sequence alignment to previously characterized reference proteomes and by identifying Pfam domains. Because of the striking differences in floral morphology and phenology between the herbaceous and woody bamboo lineages, MADS-box genes, transcription factors that control floral development and timing, were characterized and analyzed in this study. Transcripts were identified using phylogenetic methods and categorized as A, B, C, D or E-class genes, which control floral development, or *SOC* or *SVP*-like genes, which control the timing of flowering events. Putative nuclear orthologues were also identified in bamboos to use as phylogenetic markers.

**Conclusions:**

Instances of gene copies exhibiting topological patterns that correspond to shared phenotypes were observed in several gene families including floral development and timing genes. Alignments and phylogenetic trees were generated for 3,878 genes and for all genes in a concatenated analysis. Both the concatenated analysis and those of 2,412 separate gene trees supported monophyly among the woody bamboos, which is incongruent with previous phylogenetic studies using plastid markers.

**Electronic supplementary material:**

The online version of this article (doi:10.1186/s12864-016-2707-1) contains supplementary material, which is available to authorized users.

## Background

The Bambusoideae are a subfamily of perennial forest grasses endemic to every continent except Europe and Antarctica and comprise approximately 1,450 species [1, 2]. Bamboos exhibit a combination of characters that uniquely distinguish the subfamily within the larger evolutionary radiation of Poaceae. Bambusoideae is divided phylogenetically into three well-supported lineages: temperate woody (Arundinarieae), tropical woody (Bambuseae) and herbaceous (Olyreae) bamboos [2, 3]. Both lineages of woody bamboos are characterized by complex rhizome systems, a tree-like habit with highly lignified and usually hollow culms, well-differentiated culm leaves, well developed aerial branching, foliage leaf blades with outer ligules, and bisexual spikelets. Woody bamboos typically exhibit gregarious flowering cycles followed by death of the parent plants (monocarpy) [4]. They also serve as an economically important resource as they produce timber, fiber, food and other products. Herbaceous bamboos lack well differentiated culm leaves and outer ligules combined with relatively weakly lignified culms, restricted vegetative branching, unisexual spikelets and seasonal flowering [4].

Despite the uncertainty of their phylogenetic relationships [2, 3, 5–9], the two woody bamboo lineages share aspects of phenology and sexual systems suggestive of common ancestry. Their phenological patterns can be especially striking as they can exhibit extremely long intervals between flowering periods (3—120 years), which may be synchronized between disjunct populations [10]. The subsequent die-off following a flowering event can result in sudden ecological consequences such as lower shade levels in former bamboo forests.

Floral characteristics of Olyreae contrasts with those of woody bamboos in both phenology and sexual systems as the herbaceous species flower annually and possess unisexual spikelets, which are either segregated into different inflorescences or found together in a mixed inflorescence, in both cases on monoecious plants. Phenological differences between herbaceous and woody bamboos impact phylogenetic studies. Members of Olyreae generally exhibit elevated mutation rates compared to those of the woody bamboos, which are correlated with shorter generation times and longer branch lengths in phylogenetic trees [2, 11–13].

Floral development in angiosperms has been found to be largely controlled by MADS-box genes. Named after four of their homologues (*MCM1*, *AGAMOUS*, *DEFICIENS*, *SRF*), these transcription factors control the development of each of the four floral whorls (sepal, petal, stamen, and carpel). The function of MADS box genes in floral development has been extensively studied using another grass, *Oryza sativa*, as well as the eudicots *Antirrhinum majus* and *Arabidopsis thaliana*. The mechanism of development can be generally described using the classical ‘ABC’ model [14]. In this model, sepals develop with the expression of A-class genes, petals develop with the expression of A and B-class genes, stamens develop with the expression of B and C-class genes, and carpels develop with the expression of C-class genes. Subsequent studies have added D and E-class genes to the model in which expression of E-class genes is required for B and C-class function [15] and D-class gene expression is required for ovule development [16, 17] A further refinement on this understanding of floral developmental genetics is the quartet model, which suggests that MADS-box proteins work in groups of four to initiate transcription [18].

In addition to development of floral structures, the expression of some MADS box genes can affect the timing of flowering. The *SUPRRESSION OF OVER-EXPRESSION OF CONSTANS 1* (*SOC1*) gene in *A. thaliana* as well as its homolog *OsMADS56* in *O. sativa* have been characterized as being involved in several steps in the process of inducing floral development [19, 20]. The *SHORT VEGETATIVE PHASE* (*SVP*) in *A. thaliana* and *OsMADS22* + *55* in *O. sativa* have been also shown to control flowering and can act as antagonists of the *SOC1* genes [21, 22]. These genes are of particular interest in bamboos because of the aforementioned phenological characteristics. Next-generation sequencing (NGS) has allowed gene expression to be examined at a large scale for non-model organisms using the RNA-Seq method [23].  RNA-Seq typically uses mRNA selected by the poly-A tails to filter only for eukaryotic protein-coding transcripts. The RNA is reverse-transcribed into cDNA, which is fragmented and sequenced on a NGS platform such as Illumina. The RNA-Seq method had first been used in a bamboo species by Zhang et al. [24] to characterize the floral transcriptome of *Dendrocalamus latiflorus* (Bambuseae) with a follow up study on seed, leaf, stem, shoot and root tissue of the same species by Liu et al. [25]. Changes in transcript abundance in shoots of *Phyllostachys edulis* (Arundinarieae) during development were examined by Peng et al. [26] and a similar study on flowers was conducted by Gao et al. [27].

In this study, floral transcriptomes are characterized from four species representing three major bamboo lineages: *Guadua inermis* and *Otatea acuminata* (tropical woody Bambuseae), *Phyllostachys aurea* (temperate woody Arundinarieae) and *Lithachne pauciflora* (herbaceous Olyreae). The floral structure of these species vary as *L. pauciflora* has unisexual florets while the three woody bamboos have hermaphroditic florets. *P. aurea* and *O. acuminata* have three stamens [28, 29] while *G. inermis* has six. *G. inermis, O. acuminata*, and *L. pauciflora* have two stigmas [30, 31] while *P. aurea* has three [29].

These transcriptomes were analyzed to meet three complementary objectives. First, the content of each transcriptome was characterized by transcript identification and categorization. Transcripts conserved across the grass family and other plants are identified as well as bamboo-specific transcripts. Two specific methods of assembly were compared. In the past two years, the first draft nuclear genome for a bamboo (*Phyllostachys heterocycla*) was sequenced and published [32]. Our sampling scheme includes transcriptome data from the congeneric *P. aurea*, which allows for a reference-based assembly to be tested and compared to a de novo assembly.

Second, the evolutionary histories of the genes important to floral timing and development in grasses were explored. Because both bisexual and unisexual flowers and two very distinct phenological patterns are present within the taxa examined here, these includes the developmental A, B, C, D and E-class MADS-box genes as well as the *SVP* and *SOC1* gene families, which are involved in reproductive timing.

Lastly, the evolutionary history of the bamboo species examined here was examined by alignment and analysis of putative nuclear orthologues. Genes were selected based on their single-copy status in two reference taxa from the Bambusoideae-Oryzoideae-Pooideae (BOP) clade (*Brachypodium distachyon* and *O. sativa*). The goal of this portion of the study was to find markers from transcriptomic data that were available for phylogenetic analysis and to recover the overall phylogenetic signal from them. All analyses were performed on a gene-by-gene basis and also in one large concatenated alignment.

## Methods

**Fig. 1:**
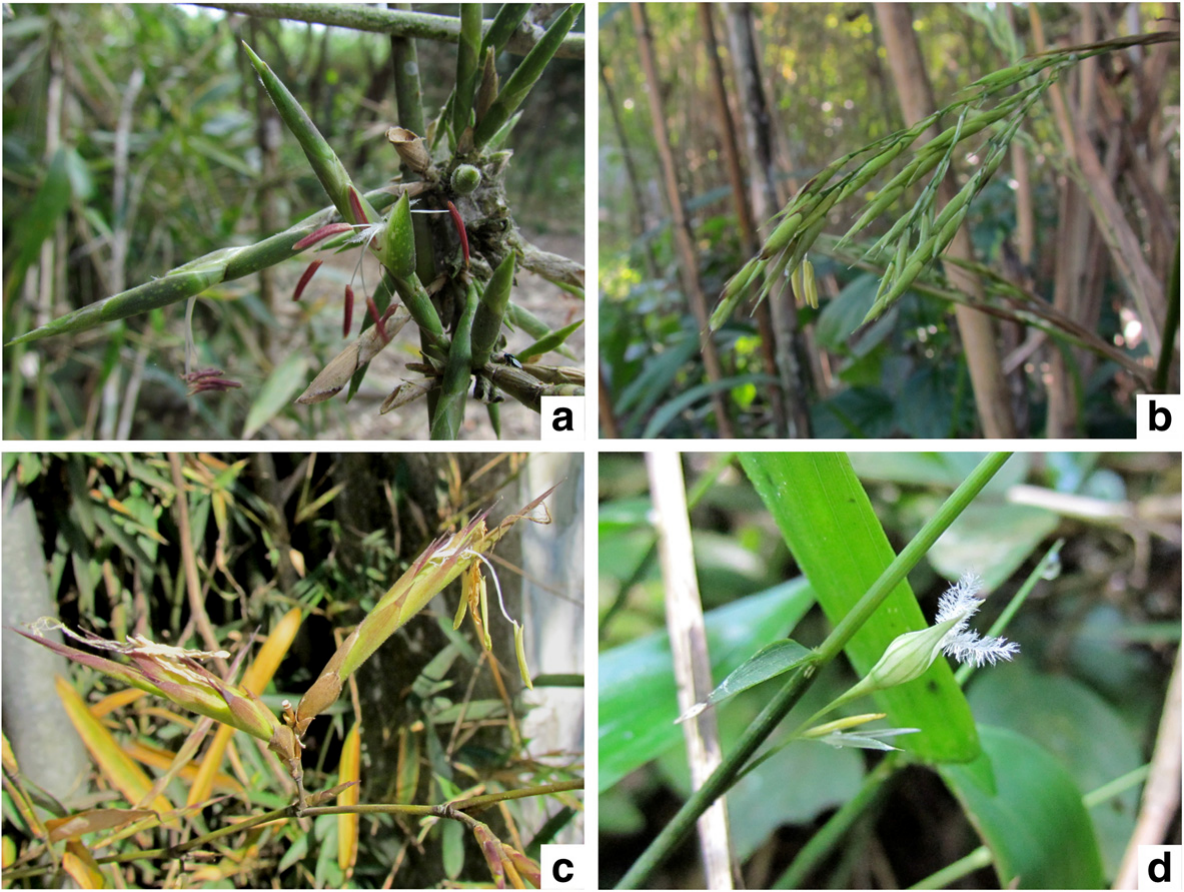
**a** *Guadua inermis* pseudospikelets. **b** *Otatea acuminata*, spikelets. **c** *Phyllostachys aurea*, pseudospikelets. **d** *Lithachne pauciflora*, male and female spikelets (floral structures of the other three species are bisexual). Note that photos (**a**, **b** and **c**) represent the actual specimens collected for this study. Photo (**d**) represents a separate individual of the same species. Photos by E. Ruiz-Sanchez

Mature spikelets from *Guadua inermis*, *Lithachne pauciflora*, *Otatea acuminata*, and *Phyllostachys aurea* were collected from tropical seasonal forests in the state of Veracruz, Mexico during the mid-dry season when florets were at full anthesis (Fig. 1). Harvested material was immediately placed in RNA-later solution (Qiagen, Valencia, CA, USA). Herbarium vouchers were collected for each species and taxonomic identities were verified (Table 1). Samples were stored at −20 °C prior to RNA extraction. Spikelets were homogenized in liquid nitrogen and a full RNA extraction and on-column DNase digestion was performed using the RNeasy extraction kit (Qiagen, Valecia, CA, USA) following the manufacturer’s protocol. Extractions were quantified with a Nanodrop 1000 (ThermoFisher Scientific, Wilmington, DE, USA) to verify a minimum concentration of 50 ng/ul. Extractions from male and female spikelets of *L. pauciflora* were pooled to represent transcripts from both genders. All other species had bisexual flowers. Library preparation and sequencing were performed at the Carver Biotechnology Center (University of Illinois, Urbana-Champaign, IL, USA). Four samples were sequenced paired-end on the HiSeq 2000 platform (Illumina, San Diego, CA, USA). Sequence areas that showed a significantly low quality score (*p* < 0.05) were deleted from each set of reads using the DynamicTrim function of SolexaQA [33]. Adapter sequences were trimmed from each read using CutAdapt v 1.7 [34].

**Table 1:** Species used in this study, with collection sites, collectors, collector numbers, the herbarium where the specimen vouchers are deposited and the number of paired-end reads generated for each specimen

Taxon	Country/state	Collectors/number	Herbarium	Number of read pairs
*Guadua inermis* Rupr. ex E. Fourn	Mexico, Veracruz	E. Ruiz-Sanchez & W. Wysocki, 466	IEB	54,488,684
*Otatea acuminata* (Munro) Calderón & Soderstr.	Mexico, Veracruz	E. Ruiz-Sanchez & W. Wysocki, 469	IEB	55,890,954
*Phyllostachys aurea* Carrière ex Rivière & C. Rivière	Mexico, Veracruz	E. Ruiz-Sanchez & W. Wysocki, 470	IEB	46,405,022
*Lithachne pauciflora* (Sw.) P. Beauv.	Mexico, Veracruz	E. Ruiz-Sanchez & W. Wysocki, 470a	IEB	58,017,553

### Transcript assembly

Two transcriptome assemblies were performed for each taxon. 1) Reads were assembled de novo into contiguous sequences (contigs) using Trinity v. r20140717 [35]. Contigs were clustered by sequence similarity using the Chrysalis function of Trinity with default settings to reduce redundancy. 2) A reference-guided assembly was performed using Tophat v. 2.0.13 [36] and Bowtie2 v. 0.12.7 [37] to map reads to the previously sequenced *Phyllostachys heterocycla* nuclear scaffolds. The parameters of Bowtie2 were optimized to account for differences between the target and reference genomes by using the ‘very-sensitive’ setting in end-to-end mode and increasing the allowed number of mismatches in each read alignment to ten. Mapped reads were then assembled using Cufflinks v. 2.2.1 [36] and the gffread function of Cufflinks was used to extract transcripts from the *P. heterocycla* genome and to identify exon boundaries. Reads were mapped to these sets of transcripts using the same parameters and the consensus sequence of each was extracted using the mpileup function of samtools [38]. Transcripts were then clustered by 100 % sequence similarity using CD-HIT v. 4.6 [39]. All subsequent analyses were performed on both sets of assembled transcripts.

TransDecoder (http://transdecoder.github.io) was used to predict putative reading frames, provide translations for each reading frame and further cluster transcripts. Putative reading frames were then screened for coding potential using CPAT v. 1.2.1 [40]. Known coding and non-coding RNA transcripts from three well-annotated plant genomes (*A. thaliana, O. sativa* and *Hordeum vulgare*) were used as training data to generate a logit model for coding potential assessment. Putative reading frames were screened for a coding potential probability over 98 %. Putative reading frames that fulfilled these coding potential criteria are henceforth referred to as putative coding transcripts (PCTs). PCTs were used as the basis of most transcriptomic quantitation in this study as they confidently reflect the protein coding assemblage and serve as a computationally efficient method of transcriptome annotation. TransDecoder was also used in combination with HMMER 3.0 [41] to predict functional domains in the PCTs using hidden Markov models (HMMs) and the Pfam database [42].

### Transcriptome content analysis

Both sets of PCTs were queried against the rice (*O. sativa*) proteome using BLASTX from the BLAST software package v. 2.2.25 [43] to identify putative function. Rice was chosen as a reference because of its phylogenetic proximity to the bamboos and high level of annotation. The BLAST query was repeated twice to screen for matches to the *A. thaliana* and *P. heterocycla* proteomes. *A. thaliana* was chosen as it has the most thoroughly explored and annotated plant genome and *P. heterocycla* was chosen to identify any bamboo-specific sequences. All PCTs that matched to at least one of these proteomes will be referred to as plant-PCTs (pPCTs).

A BLASTN query of pPCTs from the de novo assembly against those produced by the reference-based assembly was performed for each taxon with a threshold e-value of 10^−5^ and an identity cutoff of 95 % to determine which pPCTs were assembled by both methods and which were specific to their assembly method. All contigs were queried against the *Guadua weberbaueri* plastid genome (plastome) (GenBank: KP793062) using BLASTN with an e-value threshold of 10^−5^ to test for plastid contamination within the Illumina libraries.

### MADS box identification and evolutionary analysis

MADS box homologues were identified by querying those from *B. distachyon* against our respective transcript sets using BLASTP. Homologues from *B. distachyon* were used because a thorough and recent survey of the full genome was performed to identify MADS box genes [44]. Hits were filtered for redundancy and for sequences over 100 amino acids (aa) in length. Bamboo proteins were aligned conspecifically to identify copies that were assembled by both methods but differed because of assembly artifacts. Because the two most similar copies of MADS-box genes from *B. distachyon* are 93 % identical, copies from the same species that shared over 95 % identity were either reduced to one copy or merged to encompass regions of the protein that were determined using both assemblies. Bamboo sequences and previously identified homologues from *B. distachyon* and *O. sativa* were aligned using CLUSTALW [45]. Previously identified MADS-box genes from the genetically well-characterized *A. thaliana* were added to the alignment to aid in gene copy identification.

Geneious Pro v.8.1.7 (Biomatters, Auckland, New Zealand) was used to generate a neighbor-joining (NJ) tree, with 1,000 bootstrap pseudoreplicates, for all gene copies used in this study. Sequences were assigned to clades identified in Wei et al. [44] based on the presence of non-bamboo reference genes and were assigned names based on the analysis performed in Wei et al. [44]. Note that the copies from *B. distachyon* are labeled by the numbering system in Genbank rather than the numbering system in the tree generated by Wei et al. [44]. When the gene families of interest were identified, each set of protein sequences that corresponded to each family were grouped separately and outgroup OTUs were assigned based on the original NJ tree. A CLUSTALW alignment and NJ analysis was performed on each gene family separately to generate the best trees. All subsequent tree annotations were performed using the ETE Python package [46].

### Nuclear orthologue phylogenetics

Only the transcripts assembled de novo were used because they were less likely to be overrepresented based on preliminary results. Single-copy syntenic orthologous coding sequences, determined by Schnable et al. [47], were extracted from the *O. sativa* and *B. distachyon* genomes. BLASTN was used to query coding sequences from *O. sativa* against pPCTS from the four bamboo species that were assembled de novo and combined with all identified coding sequences from *B. distachyon* and *P. heterocycla*. Blast hits were filtered for a maximum e-value of 10^−5^, a minimum alignment length of 100 bp and a minimum sequence identity of 70 % following methods from Zhang et al. [48]. BLASTN results were queried for instances where one copy from *O. sativa* exhibited exactly one hit to a copy from *B. distachyon* and vice versa. The best hit from each bamboo species to each copy from *O. sativa* was located based on the highest bit-score. The homologous portions of each gene were extracted into clusters with the corresponding *O. sativa* transcript. Only groups that contained all bamboo species were used in subsequent steps. Each cluster, that included five bamboo sequences and a reference sequence from *O. sativa*, was then aligned using the LINSI algorithm of MAFFT [49], which is optimized to align nucleotide sequences accurately.

A maximum-likelihood (ML) phylogeny was then estimated using RAxML [50] for each alignment. The GTRGAMMA-I model was used for all trees. A majority consensus tree was produced from all ML trees using the Consense function in the PHYLIP software package [51]. An additional matrix was produced by concatenating all transcript alignments and removing all nucleotide positions that contained at least one gap to reduce ambiguity in downstream analyses. A ML analysis was also performed with RAxML using this matrix. The GTRGAMMA-I model was used and 1,000 ML bootstrap pseudoreplicates were performed with the concatenated alignment to assess topological support.

## Results

A total of 214,802,213 pairs of reads were sequenced for the four taxa. A total of 505,939 unique transcripts were produced from the de novo assembly and 613,319 were produced from the reference-based assembly. Open reading frame (ORF) detection followed by a coding potential filtering step in the de novo assembly yielded over 78,000 PCTs in *O. acuminata* and between 37,000 and 48,000 PCTs in the three remaining species. ORF detection in the reference-based assembly yielded between 43,000 and 105,000 PCTs. The number of reads is reported (Table 1). The number of contigs and PCTs produced for each species and method is also reported (Table 2).

**Table 2:** The number of contigs, PCTs and pPCTs generated for each taxon. Results from both assemblies are reported here

	Taxon	Contigs	PCTS	pPCTs
De novo	*G. inermis*	108438	44252	19254
	*O. acuminata*	111831	78443	31498
	*P. aurea*	88256	48001	21033
	*L. pauciflora*	197414	37848	17212
Reference	*G. inermis*	96762	43226	42788
	*O. acuminata*	180171	97094	95665
	*P. aurea*	197627	104413	103165
	*L. pauciflora*	144759	77433	76278

**Fig. 2:**
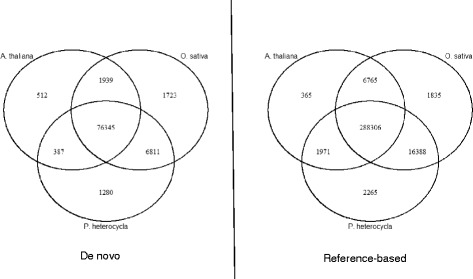
Venn diagrams reporting the number of pPCT hits that are unique to each reference proteome and shared by them. Diagrams are separated by assembly type. Venn diagrams were generated using the VennDiagram R-package

A BLASTX query of PCTs assembled de novo against the *O. sativa*, *A. thaliana*, and *P. heterocycla* proteomes (pPCTs) produced hits that covered 40–45 % of PCTs in the four bamboo species. The same query of the reference-based assembly covered > 98 % of PCTs in all four bamboo species (Table 2). The de novo assembly produced the highest number of unique pPCTs from the BLASTX search to the *O. sativa* proteome while the reference based assembly produced the highest number of unique pPCTs from the *P. heterocycla* proteome. All three genomes were able to recover 85.8 % of the pPCTs assembled de novo and 90.7 % of the pPCTs assembled using a reference genome. The numbers of pPCTs that were recovered using all reference proteomes are reported (Fig. 2).

**Fig. 3:**
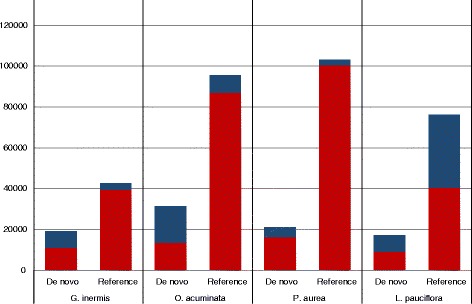
Bar graph indicating the number of pPCTs (putative transcripts that show a close plant homolog) for each taxon for both assemblies. The *red portion* of each bar indicates the number of redundant transcripts that exhibit at least 95 % nucleotide sequence identity to the other assembly. The *blue portion* represents transcripts that do not reach this criterion and are unique to their respective assembly

The BLASTN comparison between the pPCTs assembled de novo and using a reference genome revealed that in *G. inermis*, *O. acuminata* and *P. aurea* 90–98 % of pPCTs were represented in the de novo assembly by at least 95 % sequence similarity. In *L. pauciflora* 53 % of pPCTs generated using a reference genome were represented in the de novo assembly. In all taxa, 43–76 % of pPCTS assembled de novo were represented in the reference-based assemblies (Fig. 3) with the *P. aurea* assembly containing the largest portion.

**Fig. 4:**
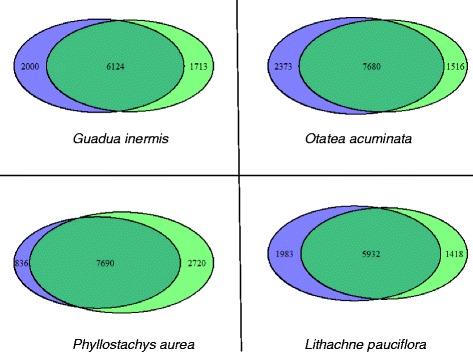
Venn diagrams reporting the number of Pfam domains that are unique to each assembly and shared by them both. Diagrams are separated by taxon. Venn diagrams are proportional to their values and were generated using the VennDiagram R-package

The HMM search on the Pfam database revealed 129,695 Pfam domains in 77,429 of the pPCTs assembled de novo (1.67 domains/pPCT) and 379,169 domains in 248,140 of the pPCTs assembled using a reference genome (1.53 domains/pPCT). A total of 13,650 unique domain types were represented in both species. The most numerous type of domain in both assemblies was the ‘protein kinase domain.’ Between 62 and 69 % of all represented Pfam domains were present in both assemblies while 7–22 % were found only in the de novo assembly and 13–25 % were only found in the reference-based assembly (Fig. 4).

Querying the contig sets for similarities to the *G. weberbaueri* full plastome produced 94 matches from transcripts assembled de novo and 101 from transcripts assembled using a reference genome. The transcripts assembled de novo ranged from 1,286–47,612 bp (see Discussion section for explanation) and those assembled using a reference ranged from 352 – 6,151 bp.

### Exploration of expressed floral genes

In the following, the terms “genes” and “gene copies” refer to those that were expressed and assembled from our transcriptomic data set. After elimination of redundant copies from the transcript sets, a total of 72 MADS-box genes were identified within the six gene families of interest: A, B, C/D, E-class, *SOC-like* and *SVP-like* MADS-box genes. The fewest number of copies were identified in the *SVP-like* gene family (4) and the most were identified in the B-class gene family (17). The fewest number of identified copies were expressed in *G. inermis* (13), while the other three taxa expressed comparable numbers (19–20). The total number of copies expressed by each taxon for all gene families is reported (Table 3). Bamboo gene copies that exhibit putative orthology to copies from *O. sativa* are listed (Additional file 1: Table S1).

**Table 3:** A total of 72 MADS-box genes were identified in this study. Numbers from each gene class and taxon are reported

Class	*G. inermis*	*O. acuminata*	*P. aurea*	*L. pauciflora*	Total
A	2	2	3	3	10
B	3	4	3	7	17
C/D	2	4	5	3	14
E	3	5	4	3	15
*SOC*-Like	2	3	4	3	12
*SVP*-Like	1	1	1	1	4
Total	13	19	20	20	72

Six neighbor-joining gene trees were generated using peptides from each of the six gene families. The gene tree topologies did not uniformly reflect previously recovered taxonomic relationships among species, but did exhibit notable patterns. A sister relationship to other bamboo species was found in 25 % of all copies from *L. pauciflora*, with the other 75 % associating either with copies from reference taxa or with clades composed of both reference and bamboo copies. A total of 55 % of copies from *P. aurea*, 69.2 % from *G. inermis*, and 73.7 % from *O. acuminata* exhibited a sister relationship to other bamboo species.

**Fig. 5:**
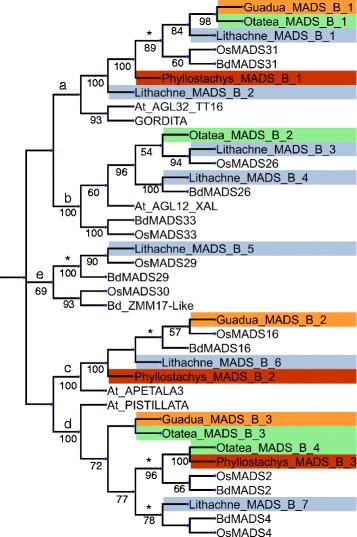
Neighbor-joining gene tree representing the B-class MADS box genes. Gene copies assembled in this study are labeled by genus, colored according to taxa (orange: *G. inermis*, green: *O. acuminata*, dark red: *P. aurea*, blue: *L. pauciflora*) and numbered redundantly to distinguish copies. Reference gene copies are not colored, are abbreviated by binomial (At: *Arabidopsis thaliana*, Bd: *Brachypodium distachyon*, Os: *Oryza sativa*) and are numbered according to their labeling in Genbank. Nodes that were supported at over 50 % bootstrap are indicated

**Fig. 6:**
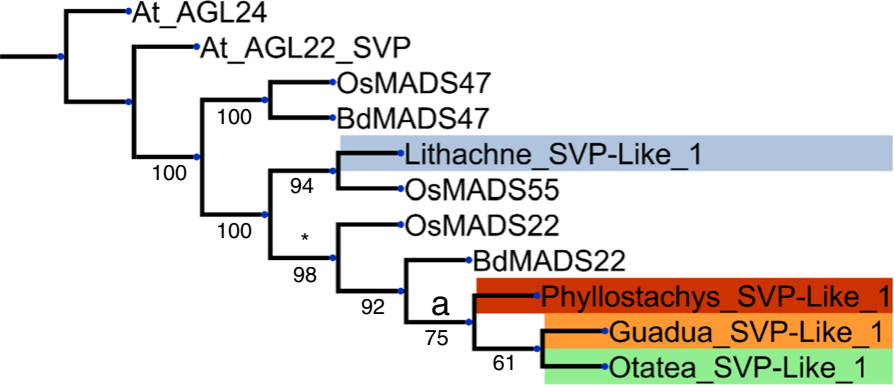
Neighbor-joining gene tree representing the *SVP*-like MADS box genes. Gene copies assembled in this study are labeled by genus, colored according to taxa (orange: *G. inermis*, green: *O. acuminata*, dark red: *P. aurea*, blue: *L. pauciflora*) and numbered redundantly to distinguish copies. Reference gene copies are not colored, are abbreviated by binomial (At: *Arabidopsis thaliana*, Bd: *Brachypodium distachyon*, Os: *Oryza sativa*) and are numbered according to their labeling in Genbank. Nodes that were supported at over 50 % bootstrap are indicated

The relationships between copies from bamboos and the reference taxa from other grass subfamilies were noted when subtrees contained representative copies from Bambusoideae, Oryzoideae (*O. sativa*) and Pooideae (*B. distachyon*) and the copies from Bambusoideae exhibited monophyly. These subtrees could be classified into three distinct categories: 1) Bamboo copies exhibit a sister relationship to copies from *O. sativa* + *B. distachyon,* 2) the copy from *B. distachyon* exhibits monophyly with bamboos and this clade exhibits a sister relationship to copies from *O. sativa,* 3) the copy from *O. sativa* exhibits monophyly with bamboos and this clade exhibits a sister relationship to copies from *B. distachyon* (see clades marked with asterisks in Figs. 5 and 6). A total of 18 distinct instances could be classified into these categories. Eight instances fell under category 1, while five instances could be classified under each of categories 2 and 3.

The B-class genes (Fig. 5) formed four clades that associate with copies of MADS-box genes from *A. thaliana* and a fifth grass-specific clade. Clade **a**, which associated with *AGL32* and *GORDITA* from *A. thaliana,* united a bamboo specific subclade with one copy each from *O. sativa* and *B. distachyon*. One additional gene copy from both *P. aurea* and *L. pauciflora* are also included in clade **a** diverging in intermediate positions. Clade **b**, which associated with *AGL12/XAL* from *A. thaliana*, comprised one copy from *O. acuminata* and two copies from *L. pauciflora.* Each of the copies from *L. pauciflora* in clade **b** formed a sister relationship to a copy from *O. sativa* or *B. distachyon*. Clade **c** associated with *APETALA1* from *A. thaliana* and contained one copy each from *G. inermis, L. pauciflora* and *P. aurea*. Clade **d** associated with *PISTILLATA* from *A. thaliana* and contained two copies from *O. acuminata*, and one from each of the other three bamboo species. Clade **d** also possessed two copies each from *O. sativa* and *B. distachyon*. The grass-specific clade **(e)** comprised one copy from *L. pauciflora*, which associated with copies *OsMADS29* and *BdMADS29*.

The A-class genes formed two distinct clades that included only grasses and one additional large clade that included grasses and *A. thaliana* (Additional file 2: Figure S1; A). Clade **a** associates with *OsMADS18* and includes one copy from *L. pauciflora, O. acuminata* and *P. aurea*, as well as a gene copy from *B. distachyon.* The second grass-specific clade (**b**) consisted of genes from only two species of grasses (*OsMADS20* + *BdMADS20*) and did not include any bamboo copies. Another grass clade associated with the *FRUITFULL*, *CAULIFLOWER*, and *APETALA1* genes from *A. thaliana* (**c**). Clade **c** included two gene copies from all bamboo species except for *O. acuminata,* which was represented by one copy.

The C/D class genes are paraphyletic and were combined into one tree (Additional file 2: Figure S1; C/D). The D-class genes from grasses form clade **a**, while another clade (**b**) includes C-class genes from grasses (subclades **c and d**), C-class genes from *A. thaliana***(e)** and one D-class gene from *A. thaliana* (*SEEDSTICK*). The four copies from *A. thaliana* formed a clade with one copy from *O. acuminata* and no other grasses (**e + f**). The remainder of the clades **(a, c, d)** were grass specific. Clade **a-1,** which associates with *OsMADS21* contains two copies from *P. aurea,* one copy from *G. inermis* and one gene copy from *L. pauciflora*. The other exclusively C-class clade **(a-2)** associates with *OsMADS13* and contains one gene copy from all bamboo species except for *G. inermis*. The next grass-specific clade of D-class genes **(c)** associated with *OsMADS3* and *BdMADS3*, contained one gene copy from all four bamboo species. The other grass-specific D-class clade **(d)** contained one gene copy from *O. acuminata,* one from *P. aurea,* two copies from *O. sativa* and one from *B. distachyon*.

The E-class gene tree (Additional file 3: Figure S2; E) formed three clades that associated with MADS-box gene copies from *A. thaliana***(a, b, c)** and one grass specific clade **(d)**. One gene copy from *O. acuminata* and one from *P. aurea* associated with *RSB* from *A. thaliana* in clade **a** along with *OsMADS6*, *BdMADS6*, and *OsMADS17*. Clade **b** contained *SEPALLATA3* from *A. thaliana,* which formed a sister relationship to two subclades of grasses that each associated with a gene copy from *O. sativa*. The first subclade **(b-1)** contained one gene copy from *G. inermis*, one copy from *L. pauciflora,* and *OsMADS8.* The second subclade **(b-2)** contained one gene copy from *O. acuminata,* one copy from *P. aurea*, *OsMADS7* and *BdMADS7*. Clade **c** contained one gene copy from *L. pauciflora* and one from *O. acuminata*, which formed a sister relationship to *SEPALLATA1 + SEPALLATA2* from *A. thaliana*. The distribution of gene copies from *O. sativa* and *B. distachyon* suggests that the grass specific clade **(d)** diverged into three subclades. The first subclade **(d-1)** contained one gene copy from *P. aurea* and associates with *OsMADS34* and *BdMADS34*. The second subclade **(d-2)** contained one gene copy from all species but *L. pauciflora* and associates with *OsMADS55* and *BdMADS55*. The third subclade **(d-3)** contains gene copies from all species except for *P. aurea* and associates with *OsMADS1* and *BdMADS1*.

The *SVP-like* gene tree (Fig. 2) contains only one copy of each bamboo species with the woody bamboos forming clade **a**. The copy from *L. pauciflora*, which is separated from the woody bamboo clade, formed a sister relationship to a copy from *O. sativa.* The *SOC-Like* gene tree (Additional file 3: Figure S2; SOC) formed a clade of grass specific genes **(a)** separate from five *A. thaliana* copies. One subclade of grasses **(b)** contained copies from all four species of bamboos, but also formed a sister relationship to a second gene copy from *L. pauciflora.* Subclade **b** also associated with *OsMADS56*, *BdMADS56* and *BdMADS50*. This subclade **(c)** contained three copies from *P. aurea*, two from *O. acuminata* and one each from *G. inermis* and *L. pauciflora.* The second subclade **(c)** is sparsely populated with bamboo copies as four copies from *B. distachyon* (*BdMADS56*, *BdMADS50*, *BdMADS7*, *BdMADS22*) are present along with two copies from *O. sativa* (*OsMADS56*, *OsMADS37*), but only seven bamboo copies, from four species, are present.

### Nuclear orthologue phylogenetics

A total of 3,878 clusters were produced that met the criteria set for this study. The length of each alignment ranged from 219 to 10,755 bp. The concatenated alignment had a length of 5,736,540 bp, which was reduced to 2,698,410 bp after gapped positions were removed.

**Fig. 7:**
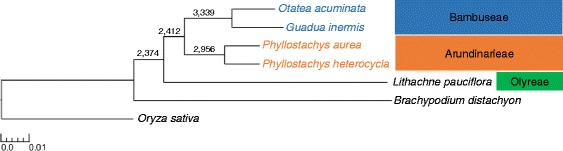
A maximum-likelihood tree generated using 3,878 concatenated gene alignments (2,698,410 bp). Branch lengths reflect number of substitution mutations. The tropical woody (Bambuseae), temperate woody (Arundinarieae) and herbaceous (Olyreae) bamboo tribes are indicated. Numbered nodes denote the number of the 3,878 best trees from each separate gene that support each node. All nodes were supported at 100 % ML bootstrap

The concatenated analysis produced a fully-resolved ML tree and was rooted at *O. sativa* based on previous phylogenetic results (Fig. 7). In this case, *B. distachyon* formed a sister relationship with the rest of the taxa (bamboos). Within Bambusoideae, *L. pauciflora* formed a sister relationship with the rest of the bamboos, which were all woody. The tropical woody bamboos, *G. inermis* + *O. acuminata* formed a sister relationship to the temperate woody bamboos, *P. aurea* + *P. heterocycla*. All relationships were supported at 100 % ML bootstrap.

Out of the 3,878 best gene trees, 2,374 trees (61.21 %) produced a monophyletic Bambusoideae when rooted at *O. sativa*. Monophyly in the woody bamboos was recovered in 2,412 trees (62.19 %). A *P. aurea* + *P. heterocycla* sister relationship was recovered in 2,956 trees (76.22 %) and a *G. inermis* + *O. acuminata* sister relationship was recovered in 3,339 trees (86.10 %). A relationship consistent with the chloroplast phylogeny was recovered in 215 trees (5.54 %).

## Discussion

### Comparison of de novo and reference-based transcriptome assemblies

While both de novo and reference-based assemblies have been used to describe full transcriptomes, these two methods, which were performed here on identical sets of reads, exhibited strikingly different transcriptomic results. The reference-based assembly produced PCT sets that were consistently shown to have higher percentages of pPCTS. This clearly reflects the nature of reference-based methods in which only reads that met a sequence similarity threshold to a previously-sequenced plant genome were assembled. All species used here exhibited either higher or comparable levels of pPCT abundance in the reference-based assembly.

The pPCTs recovered using reference proteomes from *A. thaliana*, *O. sativa* and *P. heterocycla*, could be placed into general overlapping subgroups. Those pPCTs recovered using *A. thaliana* were representative of the transcripts that could be generally found in most angiosperms given the phylogenetic distance of the eudicots to the bamboos. Those recovered using *O. sativa* were representative of genes that could be found in grasses, and those recovered using *P. heterocycla* represented genes specific to bamboos. While these taxonomic levels are not precisely defined (i.e., some hits to the *O. sativa* proteome may be indicative of BOP-clade specific transcripts), the overlap exhibits predictable patterns. In both assemblies, pPCTs that were uniquely recovered using *A. thaliana* form the smallest group while the largest group uniquely recovered in the reference assembly is from *P. heterocycla*. However, more pPCTs were uniquely recovered from *O. sativa* in the de novo assembly. This could be indicative of a weakness in the de novo assembly in which transcripts are present, but not at high enough abundances to assemble them without the aid of a reference genome. It also could be an artifact of the reference-based assembly and reliance on the *P. heterocycla* genome. The lower representation level in the de novo assembly may be due to its incompleteness.

When a 95 % sequence identity threshold was used to assess redundancy, a much larger portion of the pPCTs assembled de novo is unique to each assembly, except those from *L. pauciflora*. For the three woody species, this would indicate that the de novo assembly produced a set of unique transcripts while the reference based assembly produced transcripts that were largely represented in the de novo assembly. This is likely indicative of under-assembly in the reference-based trancripts. While this could be an indication that a reference-based assembly is largely unnecessary and could artificially inflate the number of transcripts produced, the number of uniquely represented transcripts in the reference-based *L. pauciflora* assembly may suggest otherwise.

The reference-based assembly likely recovered a large number of transcripts in *L. pauciflora* with coverage too low to be assembled de novo. This does not reflect any inherent property of the *L. pauciflora* reads (lower number, quality, etc.) but may reflect a difference in genomic properties. While exact ploidy levels are not known for the species analyzed in this study, herbaceous bamboos are known to be primarily diploid, while woody bamboos tend to exhibit higher levels of ploidy likely resulting from hybridizations [9, 52, 53]. A lower ploidy level in *L. pauciflora* could have been conducive to a larger spread of transcriptomic representation due to higher coverage levels for each gene.

Further support for performing both types of assembly comes from the Pfam analysis in the pPCTs. Only 62-69 % of all represented Pfam domains were present in both assemblies even though both shared a considerable proportion of unique protein domains (Fig. 4). The reference-based assembly for *P. aurea* produced more than three times the number of unique Pfam domains than the de novo assembly. While the other three produced fewer, this is most likely due to the phylogenetic proximity of *P. aurea* to the congeneric reference genome [54].

### Presence of plastid genome sequences

Although the Illumina libraries were sequenced after selection for transcripts containing poly-A tails, sequences exhibiting plastome homology were likely assembled due to the high number of plastids present in each plant cell. The presence of plastid sequences may have been retained during the poly-A selection step due to the AT richness of the plastome. A large number of these transcripts, which were assembled de novo, were much larger than mRNA transcripts that were observed in eukaryotic plastomes > 10 kbp and contained more than one coding sequence. One explanation for these unusually large transcripts is that plastid genomes are typically very compact and contain relatively small intergenic regions. If overlapping UTRs were present in the sequenced transcripts, it could be an artifact of the de novo assembly method, which takes no reference genome into account and produces assemblies based solely on sequence identity.

### Floral gene analysis

Because the field-harvested sources of our RNA extracts were spikelets with fully developed and emergent florets, not all MADS-box genes (A-E class) were expected to be expressed. This is apparent as significantly fewer MADS box genes were found in each bamboo transcriptome (13–20) than the 57 that were found in the fully sequenced *B. distachyon* genome [44]. Between two and five genes were identified in all classes for each taxon except for *L. pauciflora,* which expressed seven distinct B-class genes. Because B-class genes are important in lodicule and stamen development in grasses [55], they are required in the development of both types of unisexual florets that are produced by herbaceous bamboos. We hypothesize that duplications of two B class genes may have allowed separate copies to be expressed differentially in either male (lodicule and stamen development) or female (lodicule only development) florets. Though *O. sativa* and *B. distachyon* have hermaphroditic florets and are both represented with seven copies, their data is genomic and the lower number of transcripts in the three woody bamboos is likely a result of differential expression. The first of these, involving gene copies B1 and B2, are both found in clade **a** (Fig. 5). Gene copy B1 clusters with (*OsMADS31* + *BdMADS31*). Gene copy B2 is sister to all of the grass-specific members of this clade, rather than to any specific gene copy, although copy B1 of *P. aurea* is the next copy in the clade to diverge. In the second case there are two copies, B3 and B4, which are found in clade **b** (Fig. 5) and cluster with *OsMADS26* and *BdMADS26*, respectively. Unexpectedly copy B5 from *L. pauciflora* does not cluster with any other copies from Bambusoideae, so its origin is obscure. This hypothesis could be verified by sequencing B-class genes from separate staminate and carpellate florets.

In several cases, correlations between gene copy number and differences in floral phenotypes can be hypothesized. The *PISTILLATA* homologues from *O. sativa*, *OsMADS2* and *OsMADS4* were shown to have complementary importance in lodicule development and about equal importance in stamen development [56]. One copy each from *O. acuminata* and *P. aurea* associate with *OsMADS2* (Fig. 5; clade d), which may be indicative of phenotype as both have three stamens compared to *G. inermis*, which has six stamens [57]. However, sister to the *OsMADS2-4* subclade within clade d are one copy each from *G. inermis* and *O. acuminata* which may be indicative of a duplication resulting in a novel, bamboo-specific, B-class gene. Another potentially bamboo-specific gene can be found in one copy from *L. pauciflora* and *O. acuminata* to the E-class *SEPALLATA1-2* genes from *A. thaliana* (Additional file 3 Figure S2; E-clade c). The placement of these copies seems to be indicative of a gene duplication in bamboos or a copy deletion in non-bamboo grasses.

Within the C/D-class clade, which are paraphyletic in regard to function as previously noted in broad studies of fruit development genes [58], *OsMADS3* and *OsMADS58* are known to be C-class genes. *OsMADS3* has been shown to be important in carpel diversification [59] and clusters with copies from *G. inermis*, *O. acuminata*, and *L. pauciflora* (Additional file 2: Figure S1; C/D-clade c). These three genes also distantly associate to a copy from *P. aurea*. This could potentially be indicative of the phenotypic difference in carpels between the clade formed by copies from *G. inermis*, *O. acuminata. L. pauciflora, O. sativa*, and B*. distachyon*, which have two stigmas, and *P. aurea*, which has three stigmas and forms a sister relationship with the two-stigma clade.

Another potential connection to phenotype can be found in the gene tree for *SVP-like* genes, which has been tied to flowering time. One copy from each species of bamboo was present in this tree (Fig. 6). The copy from *L. pauciflora* is the immediate sister to a copy from *O. sativa*, which also flowers annually, rather than with the other three bamboo species, the latter of which formed clade **a** and exhibited 92.6 % sequence similarity. The copy from *L. pauciflora* exhibited between 69 and 82 % sequence similarity to the other bamboo copies. This may be explained by the different phenological patterns found among these species; *L. pauciflora* is a perennial that flowers approximately annually while the other three taxa flower at very long intervals [4].

These phenotypic connections to expressed gene copy number and evolutionary history are interesting, and could be confirmed with subsequent testing. In situ hybridization and transcriptome sampling at different stages of floral development could be performed to verify these hypotheses. The bioinformatics-based survey performed in this study is a foundation to further elucidating the complete flowering mechanisms of these, and other, bamboo species.

The presence of sister orthologues of *O. sativa* and *B. distachyon* that do not associate with bamboo copies is observed at least three times (Fig. 5, clade b: *OsMADS33-BdMADS33;* clade e: *OsMADS30-Bd_ZMM17-Like;* Additional file 2: Figure S1, clade b: *OsMADS20-BdMADS20*). The most probably explanation for this pattern is that orthologues (or close homologues) of these genes were not expressed in the four tissues that were used in this study. This is especially likely as some A/B-class genes are known to be expressed earlier in floral development and the florets harvested for this study were fully developed. The second possibility is that there was a deletion of these copies in the Bambusoideae. The possibility of gene duplication in the *B. distachyon* and *O. sativa* lineage(s) is very unlikely as previous phylogenetic studies have placed the Bambusoideae either sister to *B. distachyon* or sister to *O. sativa* (see below).

One important caveat to any of the transcriptome comparisons made within this study is that inconsistencies may arise from the method of tissue retrieval and the study system used. While the floral tissue was harvested from spikelets of approximately equal maturity, the stresses and conditions endured by each plant (i.e., soil type, climate, herbivory) may have been significantly different. This method of collecting floral tissue is necessary when using bamboos as a study system since their flowering cycles are typically very long and unpredictable, and they are difficult to cultivate as flowering specimens under greenhouse conditions with few exceptions [60]. Genome sequencing followed by an extensive survey for functional genes would allow us to more confidently confirm the presence or absence of specific gene copies.

### Floral genes and phylogeny

The repeated instances of gene copies from *L. pauciflora* being isolated from copies from other bamboos in our gene trees (Figs. 5 and 6) could be explained by the differences between Olyreae and the two woody bamboo tribes (Arundinarieae and Bambuseae) in phenology, sexual systems, floral development and structure. This could be an indication that Olyreae are evolutionarily separate from the woody bamboo lineages, but contradicts previous studies that have placed Olyreae in a sister relationship to Bambuseae [2, 13, 61]. This potential indication of shared ancestry between Arundinarieae and Bambuseae is compatible with the results of Triplett et al. [9], who proposed a monophyletic woody bamboo clade (Bambuseae + Arundinarieae) on the basis of phylogenetic analysis of single-copy nuclear markers. However, complete genomic sequencing and annotation would be required to rule out the possibility that these topologies are a result of unexpressed, and therefore missing, copies.

Most of the subfamilial grass phylogeny that could be inferred from these trees showed a topology in which bamboos are more closely related to *O. sativa* than to *B. distachyon*. This contrasts markedly with previously published phylogenetic studies that use plastid markers [2, 13, 61], which place the Oryzoideae (*O. sativa*) in a sister relationship to the Bambusoideae + Pooideae (*B. distachyon*) clade. However, some studies that used plastid markers have placed Bambusoideae in a sister relationship to Oryzoideae [7, 62].

The use of nuclear transcripts as phylogenetic markers has been controversial because of the shorter length of single transcripts, selective effects on coding mRNAs and the ambiguity in differentiating orthologues from paralogues (or other homologues). The aforementioned speculations based on MADS box gene tree topological patterns can be tested extensively, using a large variety of nuclear markers, to draw robust conclusions about the evolutionary relationships among these taxa.

### Nuclear orthologue phylogenetics

The recovery of two conflicting tribal topologies in the Bambusoideae brings two main questions into consideration: 1) Which topology reflects the actual species tree and evolutionary history of Bambusoideae? 2) Which evolutionary events would cause these conflicts to emerge? The trees produced using concatenated nuclear genes strongly supported the hypothesis of a woody bamboo clade (Fig. 7). A monophyletic relationship among the woody bamboos is also supported in the majority of separate gene trees. We will refer to this topology, ((Arundinarieae + Bambuseae), Olyreae), as the ‘nuclear hypothesis’ of bamboo evolution. Phylogenetic trees that use complete sets of coding and noncoding regions from plastid genomes have recovered a well-supported paraphyly in the woody bamboos, ((Olyreae + Bambuseae), Arundinarieae), which we will refer to as the ‘plastid hypothesis.’

Based purely on the quantity of data, the nuclear hypothesis is supported by over an order of magnitude more than the plastid hypothesis. The nuclear hypothesis is supported by the phylogenetic signal given by over 3,700 loci, while the complete plastid genome is inherited cytoplasmically and theoretically gives the phylogenetic signal equivalent to one locus. However, the single-locus attribute of the plastid genome gives it a much higher degree of certainty in its use as a phylogenetic marker between plant species. Although care was taken to minimize our level of uncertainty, nuclear genes can have multiple paralogous copies. Their use as phylogenetic markers can also be complicated by allopolyploidy, which many grasses have been shown to exhibit [63].

Although the use of morphological characteristics in the determination of phylogenetic relatedness is controversial, the morphological and phenological characteristics of the three bamboo tribes are notable. Arundinarieae and Bambuseae share a suite of characteristics with highly lignified shoots, bisexual flowers and intermittent flowering events followed by a die-off. Olyreae have shoots with significantly less lignification, unisexual florets and annual flowering. The nuclear hypothesis suggests a single origin of these characteristics and the plastid hypothesis suggests two origins or one origin followed by a loss of these characteristics in Olyreae. The duplicate origin of similar characteristics seems unlikely, but a loss of certain characters could be biologically feasible.

A hypothesis for the validity of both phylogenetic signals involves an ancient hybridization. With the species tree following the nuclear hypothesis, the Bambuseae and Arundinarieae would exhibit a sister relationship to Olyreae. If a progenitor species of Bambuseae had hybridized with a sympatric progenitor species of Olyreae, followed by a back-cross in the paternal species, the maternally inherited plastid signal would place Bambuseae phylogenetically sister to Olyreae rather than Arundinarieae. An alternative explanation for the incongruence of phylogenetic signal could be selection in which either tropical bamboos (Bambuseae and Olyreae) or woody bamboos (Bambuseae and Arundinarieae) accumulated homoplasious mutations. However, it would be very unlikely that over 60 % of the genes sampled in our analyses skewed the phylogenetic signal identically, placing Bambuseae sister to Arundinarieae, due to selection. Long-branch attraction can be eliminated as a possibility because both Bambuseae and Arundinarieae produce very short branches within each tribe [13].

Triplett et al. [9] also recovered monophyly in the woody bamboos using three low copy nuclear genes. However, each gene copy was classified into respective ancient genomes based on the hypothesis of woody bamboos being a product of allopolyploidization. Our study assumed orthology based on the highest sequence similarity and the presence of one orthologous copy per species. While the single-orthologue approach does not account for multiple orthologous copies from allopolyploids, the overall phylogenetic signal from the concatenated alignment and trees is robust. If the putative orthologues from each taxon originated from different progenitor woody bamboo genomes, we might expect the support for nodes within the woody bamboo clade to reflect this.

## Conclusions

This marks the first study that compared transcriptomes across Bambusoideae and the first transcriptome generated from an herbaceous bamboo. Methodologically it was demonstrated that transcriptome assembly can be performed using de novo or reference-based methods. De novo methods may have over-assembled the reads (in that more transcripts in vivo were represented by fewer transcripts *in silico*) while reference-based methods may have under-assembled (in which more transcripts were produced *in silico* that represented fewer in vivo). This study also identified expressed MADS-box genes in the bamboo species represented here. Although these genes do not represent an exhaustive genomic survey, they can be used in futures studies to thoroughly examine bamboo floral development. The lack of phylogenetic information in MADS-box genes was also clarified, at least in bamboos. The phylogenetic utility of a full transcriptome was demonstrated by identifying putative orthologues in each transcriptome and performing a maximum likelihood analysis. The origin of woody bamboos was supported as monophyletic, which contrasted with many studies that used plastid markers. Sequencing of full nuclear genomes would be required to confirm orthology although it is unlikely that most gene analyses identically produced erroneous results.

## Availability of data and materials

All reads were deposited into the SRA database at the NCBI and can be accessed under the experimental accession SRX1553102.

## Additional files

Additional file 1: Table S1: MADS-box gene copies from bamboos matched to corresponding orthologs from *Oryza sativa*. MADS-box gene classes are noted. Gene copies in parentheses denote orthologs that were inferred ambiguously based on topological relationships. (DOC 58 kb) Additional file 2: Figure S1: Neighbor-joining gene tree representing the A and C/D-class MADS box genes. Gene copies assembled in this study are labeled by genus, colored according to taxa (orange: *G. inermis*, green: *O. acuminata*, dark red: *P. aurea*, blue: *L. pauciflora*) and numbered redundantly to distinguish copies. Reference gene copies are not colored, are abbreviated by binomial (At: *Arabidopsis thaliana*, Bd: *Brachypodium distachyon*, Os: *Oryza sativa*) and are numbered according to their labeling in Genbank. Nodes that were supported at over 50 % bootstrap are indicated. (PDF 331 kb) Additional file 3: Figure S2: Neighbor-joining gene tree representing the SOC-like and E-class MADS box genes. Gene copies assembled in this study are labeled by genus, colored according to taxa (orange: *G. inermis*, green: *O. acuminata*, dark red: *P. aurea*, blue: *L. pauciflora*) and numbered redundantly to distinguish copies. Reference gene copies are not colored, are abbreviated by binomial (At: *Arabidopsis thaliana*, Bd: *Brachypodium distachyon*, Os: *Oryza sativa*) and are numbered according to their labeling in Genbank. Nodes that were supported at over 50 % bootstrap are indicated. (PDF 465 kb)

## References

[1] Bamboo Phylogeny Group [BPG]. An updated tribal and subtribal classification of the bamboos (Poaceae: Bambusoideae). In: Gielis J, Potters G, editors. P 9th World Bamboo Congr. American Bamboo Society. Antwerp; 2012. p. 3–27.

[2] Kelchner SA, Bamboo Phylogeny Group (2013). Higher level phylogenetic relationships within the bamboos (Poaceae: Bambusoideae) based on five plastid markers. Mol Phylogenet Evol.

[3] Sungkaew S, Stapleton CMA, Salamin N, Hodkinson TR (2009). Non-monophyly of the woody bamboos (Bambuseae; Poaceae): a multi-gene region phylogenetic analysis of Bambusoideae s.s. J Plant Res.

[4] Clark LG, Londoño X, Ruiz-Sanchez E. Bamboo taxonomy and habitat. In: Laslo P, Köehl M, (eds). Bamboo. Series: Tropical Forestry Handbook. Springer. 2015. (In press)

[5] Zhang WP (2000). Phylogeny of the Grass Family (Poaceae) from rpl16 Intron Sequence Data. Mol Phylogenet Evol.

[6] Zhang WP, Clark LG, Jacobs SWL, Everett J (2000). Phylogeny and classification of the Bambusoideae (Poaceae). Grass Syst Evol.

[7] Grass Phylogeny Working Group [GPWG] (2001). Phylogeny and subfamilial classification of the grasses (Poaceae). Ann Missouri Bot Gard.

[8] Bouchenak-Khelladi Y, Salamin N, Savolainen V, Forest F, van der Bank M (2008). Large multi-gene phylogenetic trees of the grasses (Poaceae): progress towards complete tribal and generic level sampling. Mol Phylogenet Evol.

[9] Triplett JK, Clark LG, Fisher AE, Wen J (2014). Independent allopolyploidization events preceded speciation in the temperate and tropical woody bamboos. New Phytol.

[10] Janzen DH (1976). Why bamboos wait so long to flower. Ann Rev Ecol Syst.

[11] Gaut BS, Clark LG, Wendel JF, Muse SV (1997). Comparisons of the molecular evolutionary process at *rbcL* and *ndhF* in the grass family (Poaceae). Mol Biol Evol.

[12] Oliveira RP, Clark LG, Schnadelbach AS, Monteiro SH, Borba EL (2014). A molecular phylogeny of *Raddia* and its allies within the tribe Olyreae (Poaceae, Bambusoideae) based on noncoding plastid and nuclear spacers. Mol Phylogenet Evol.

[13] Wysocki WP, Clark LG, Attigala L, Ruiz-Sanchez E, Duvall MR (2015). Evolution of the bamboos (Bambusoideae; Poaceae): a full plastome phylogenomic analysis. BMC Evol Biol.

[14] Coen ES, Meyerowitz EM (1991). The war of the whorls: genetic interactions controlling flower development. Nature.

[15] Pelaz S, Ditta GS, Baumann E, Wisman E, Yanofsky MF. B and C floral organ identity functions require *SEPALLATA* MADS-box genes. Nature. 2000;405(6783):200–3. 10.1038/35012103 10821278

[16] Skinner DJ, Hill TA, Gasser CS (2004). Regulation of ovule development. Plant Cell.

[17] Dreni L, Jacchia S, Fornara F, Fornari M, Ouwerkerk PB (2007). The D‐lineage MADS‐box gene *OsMADS13* controls ovule identity in rice. Plant J.

[18] Theißen G, Saedler H (2001). Plant biology: floral quartets. Nature.

[19] Ryu CH, Lee S, Cho LH, Kim SL, Lee YS (2009). *OsMADS50* and *OsMADS56* function antagonistically in regulating long day (LD)‐dependent flowering in rice. Plant Cell Environ.

[20] Lee J, Lee I (2010). Regulation and function of *SOC1*, a flowering pathway integrator. J Exp Bot.

[21] Hartmann U, Höhmann S, Nettesheim K, Wisman E, Saedler H, Huijser P (2000). Molecular cloning of SVP: a negative regulator of the floral transition in Arabidopsis. Plant J.

[22] Lee JH, Park SH, Ahn JH (2012). Functional conservation and diversification between rice *OsMADS22*/*OsMADS55* and *Arabidopsis SVP* proteins. Plant Sci.

[23] Wang Z, Gerstein M, Snyder M (2009). RNA-Seq: a revolutionary tool for transcriptomics. Nature Rev Genet.

[24] Zhang XM, Zhao L, Larson-Rabin Z, Li DZ, Guo ZH (2012). De novo sequencing and characterization of the floral transcriptome of *Dendrocalamus latiflorus* (Poaceae: Bambusoideae). PLoS One.

[25] Liu M, Qiao G, Jiang J, Yang H, Xie L (2012). Transcriptome sequencing and de novo analysis for ma bamboo (*Dendrocalamus latiflorus* Munro) using the Illumina platform. PLoS One.

[26] Peng Z, Zhang C, Zhang Y, Hu T, Mu S (2013). Transcriptome sequencing and analysis of the fast growing shoots of Moso bamboo (*Phyllostachys edulis*). PloS One.

[27] Gao J, Zhang Y, Zhang C, Qi F, Li X (2014). Characterization of the Floral Transcriptome of Moso Bamboo (*Phyllostachys edulis*) at Different Flowering Developmental Stages by Transcriptome Sequencing and RNA-Seq Analysis. PLoS One.

[28] Ruiz-Sanchez E, Sosa V, Mejía-Saules MT, Londoño X, Clark LG (2011). A taxonomic revision of *Otatea* (Poaceae: Bambusoideae: Bambuseae) including four new species. Syst Bot.

[29] Kellogg EA. V. Subfamily Bambusoideae Luerss (1893). In: Flowering Plants. Monocots. Switzerland: Springer International Publishing; 2015. p. 151–98.

[30] Kellogg EA. IV. Subfamily Ehrhartoideae Link (1827). In: Flowering Plants. Monocots. Switzerland: Springer International Publishing; 2015. p. 143–50.

[31] Kellogg EA. VI. Subfamily Pooideae Benth (1861). In: Flowering Plants. Monocots. Switzerland: Springer International Publishing; 2015. p. 199–265.

[32] Peng Z, Lu Y, Li L, Zhao Q, Feng Q (2013). The draft genome of the fast-growing non-timber forest species moso bamboo (*P. heterocycla*). Nature Genet.

[33] Cox MP, Peterson DA, Biggs PJ (2010). SolexaQA: At-a-glance quality assessment of Illumina second-generation sequencing data. BMC Bioinformatics.

[34] Martin M (2011). Cutadapt removes adapter sequences from high-throughput sequencing reads. EMBnet J.

[35] Grabherr MG, Haas BJ, Yassour M, Levin JZ, Thompson DA (2011). Full-length transcriptome assembly from RNA-seq data without a reference genome. Nat Biotech.

[36] Trapnell C, Pachter L, Salzberg SL (2009). TopHat: discovering splice junctions with RNA-Seq. Bioinformatics.

[37] Langmead B, Salzberg SL (2012). Fast gapped-read alignment with Bowtie 2. Nat Methods.

[38] Li H, Handsaker B, Wysoker A, Fennell T, Ruan J (2009). The sequence alignment/map format and SAMtools. Bioinformatics.

[39] Li W, Godzik A (2006). Cd-hit: a fast program for clustering and comparing large sets of protein or nucleotide sequences. Bioinformatics.

[40] Wang L, Park HJ, Dasari S, Wang S, Kocher JP (2013). CPAT: Coding-Potential Assessment Tool using an alignment-free logistic regression model. Nucleic Acids Res.

[41] Finn RD, Clements J, Eddy SR (2011). HMMER web server: interactive sequence similarity searching. Nucleic Acids Res.

[42] Bateman A, Coin L, Durbin R, Finn RD, Hollich V (2004). The Pfam protein families database. Nucleic Acids Res.

[43] Altschul SF, Madden TL, Schäffer AA, Zhang J, Zhang Z (1997). Gapped BLAST and PSI-BLAST: a new generation of protein database search programs. Nucleic Acids Res.

[44] Wei B, Zhang RZ, Guo JJ, Liu DM, Li AL (2014). Genome-wide analysis of the MADS-box gene family in *Brachypodium distachyon*. PLoS One.

[45] Thompson JD, Higgins DG, Gibson TJ (1994). CLUSTAL W: improving the sensitivity of progressive multiple sequence alignment through sequence weighting, position-specific gap penalties and weight matrix choice. Nucleic Acids Res.

[46] Huerta-Cepas J, Dopazo J, Gabaldón T (2010). ETE: a python Environment for Tree Exploration. BMC Bioinformatics.

[47] Schnable JC, Freeling M, Lyons E (2012). Genome-wide analysis of syntenic gene deletion in the grasses. Genome Biol Evol.

[48] Zhang LN, Zhang XZ, Zhang YX, Zeng CX, Ma PF, Zhao L, Guo ZH, Li DZ (2014). Identification of putative orthologous genes for the phylogenetic reconstruction of temperate woody bamboos (Poaceae: Bambusoideae). Mol Ecol Resources.

[49] Katoh K, Standley DM (2013). MAFFT multiple sequence alignment software version 7: improvements in performance and usability. Mol Biol Evol.

[50] Stamatakis A (2006). RAxML-VI-HPC: maximum likelihood-based phylogenetic analyses with thousands of taxa and mixed models. Bioinformatics.

[51] Felsenstein J. PHYLIP Seattle: Department of Genome Science. Seattle, WA: University of Washington; 2005. p. 3.

[52] Hunziker JH, Wulff AF, Soderstrom TR (1982). Chromosome studies on the Bambusoideae (Gramineae). Brittonia.

[53] Chokthaweepanich H. Phylogenetics and evolution of the paleotropical woody bamboos (Poaceae: Bambusoideae: Bambuseae). Dissertation. Ames, IA: Iowa State University; 2014.

[54] Triplett JK, Clark LG (2010). Phylogeny of the Temperate Bamboos (Poaceae: Bambusoideae: Bambuseae) with an emphasis on *Arundinaria* and Allies. Syst Bot.

[55] Whipple CJ, Ciceri P, Padilla CM, Ambrose BA, Bandong SL, Schmidt RJ (2004). Conservation of B-class floral homeotic gene function between maize and *Arabidopsis*. Development.

[56] Yao SG, Ohmori S, Kimizu M, Yoshida H (2008). Unequal genetic redundancy of rice PISTILLATA orthologs, OsMADS2 and OsMADS4, in lodicule and stamen development. Plant Cell Physiol.

[57] Judziewicz EJ, Clark LG, Londoño X, Stern MJ. American bamboos. Washington D.C.: Smithsonian Institution Press; 1999.

[58] Pabón-Mora N, Wong GS, Ambrose BA (2014). Evolution of fruit development genes in flowering plants. Frontiers in plant science.

[59] Yamaguchi T, Lee DY, Miyao A, Hirochika H, An G, Hirano HY (2006). Functional diversification of the two C-class MADS box genes *OSMADS3* and *OSMADS58* in *Oryza sativa*. Plant Cell.

[60] Lin CS, Lin CC, Chang WC (2005). Shoot regeneration, re-flowering and post flowering survival in bamboo inflorescence culture. Plant Cell Tiss Org Cult.

[61] Wu ZQ, Ge S (2012). The phylogeny of the BEP clade in grasses revisited: evidence from the whole-genome sequences of chloroplasts. Mol Phylogenet Evol.

[62] Clark LG, Zhang W, Wendel JF (1995). A phylogeny of the grass family (Poaceae) based on ndhF sequence data. Syst Bot.

[63] Levy AA, Feldman M (2002). The impact of polyploidy on grass genome evolution. Plant Physiol.

