# Supplementary material for: The floral transcriptomes of four bamboo species (Bambusoideae; Poaceae): support for common ancestry among woody bamboos
Source: BMC Genomics. 2016 May 20;17:384. doi: 10.1186/s12864-016-2707-1 (PMC4875691; doi:10.1186/s12864-016-2707-1)
Supplement: Additional file 1: Table S1: — MADS-box gene copies from bamboos matched to corresponding orthologs from Oryza sativa. MADS-box gene classes are noted. Gene copies in parentheses denote orthologs that were inferred ambiguously based on topological relationships. (DOC 58 kb) [file 12864_2016_2707_MOESM1_ESM.doc]

| Gene Class | *Oryza sativa* | *Guadua inermis* | *Otatea acuminata* | *Phyllostachys aurea* | *Lithachne pauciflora* |
| --- | --- | --- | --- | --- | --- |
| A | *OsMADS14* | Guadua_MADS_A_2 | - | Phyllostachys_MADS_A_3 | (Lithachne_MADS_A_3) |
|  | *OsMADS15* | - | - | Phyllostachys_MADS_A_2 | Lithachne_MADS_A_2 |
|  | *OsMADS18* | - | Otatea_MADS_A_1 | Phyllostachys_MADS_A_1 | Lithachne_MADS_A_1 |
|  | *OsMADS20* | - | - | - | - |
| B | *OsMADS2* | - | Otatea_MADS_B_4 | Phyllostachys_MADS_B_3 | - |
|  | *OsMADS4* | - | - | - | Lithachne_MADS_B_7 |
|  | *OsMADS16* | Guadua_MADS_B_2 | - | Phyllostachys_MADS_B_2 | Lithachne_MADS_B_6 |
|  | *OsMADS26* | - | Otatea_MADS_B_2 | - | Lithachne_MADS_B_3,4 |
|  | *OsMADS29* | - | - | - | Lithachne_MADS_B_5 |
|  | *OsMADS30* | - | - | - | - |
|  | *OsMADS31* | Guadua_MADS_B_1 | Otatea_MADS_B_1 | Phyllostachys_MADS_B_1 | Lithachne_MADS_B_1,2 |
|  | *OsMADS33* | - | - | - | - |
| C/D | *OsMADS3* | Guadua_MADS_C/D_2 | Otatea_MADS_C/D_3 | Phyllostachys_MADS_C/D_4 | Lithachne_MADS_C/D_3 |
|  | *OsMADS13* | - | Otatea_MADS_C/D_2 | Phyllostachys_MADS_C/D_3 | Lithachne_MADS_C/D_2 |
|  | *OsMADS21* | Guadua_MADS_C/D_1 | - | Phyllostachys_MADS_C/D_1,2 | Lithachne_MADS_C/D_1 |
|  | *OsMADS58* | - | (Otatea_MADS_C/D_4) | (Phyllostachys_MADS_C/D_5) | - |
|  | *OsMADS66* | - | (Otatea_MADS_C/D_4) | (Phyllostachys_MADS_C/D_5) | - |
| E | *OsMADS1* | Guadua_MADS_E_3 | Otatea_MADS_E_1 | - | Lithachne_MADS_E_2 |
|  | *OsMADS5* | Guadua_MADS_E_2 | Otatea_MADS_E_3 | Phyllostachys_MADS_E_4 | - |
|  | *OsMADS6* | - | Otatea_MADS_E_2 | Phyllostachys_MADS_E_2 | - |
|  | *OsMADS7* | - | Otatea_MADS_E_1 | Phyllostachys_MADS_E_1 | - |
|  | *OsMADS8* | Guadua_MADS_E_1 | - | - | Lithachne_MADS_E_1 |
|  | *OsMADS17* | - | - | - | - |
|  | *OsMADS34* | - | - | Phyllostachys_MADS_E_3 | - |
| *SOC* | *OsMADS37* | - | Otatea_SOC-Like_3 | Phyllostachys_SOC-Like_3 | - |
|  | *OsMADS56* | Guadua_SOC-Like_1 | Otatea_SOC-Like_1 | Phyllostachys_SOC-Like_1 | Lithachne_SOC-Like_1,2 |
|  | *OsMADS65* | Guadua_SOC-Like_2 | Otatea_SOC-Like_2 | Phyllostachys_SOC-Like_2 | Lithachne_SOC-Like_3 |
| *SVP* | *OsMADS22* | Guadua_SVP-Like_1 | Otatea_SVP-Like_1 | Phyllostachys_SVP-Like_1 | - |
|  | *OsMADS47* | - | - | - | - |
|  | *OsMADS55* | - | - | - | Lithachne_SVP-Like_1 |
