# Supplementary figures and images for: The floral transcriptomes of four bamboo species (Bambusoideae; Poaceae): support for common ancestry among woody bamboos
Source: BMC Genomics. 2016 May 20;17:384. doi: 10.1186/s12864-016-2707-1 (PMC4875691; doi:10.1186/s12864-016-2707-1)

A

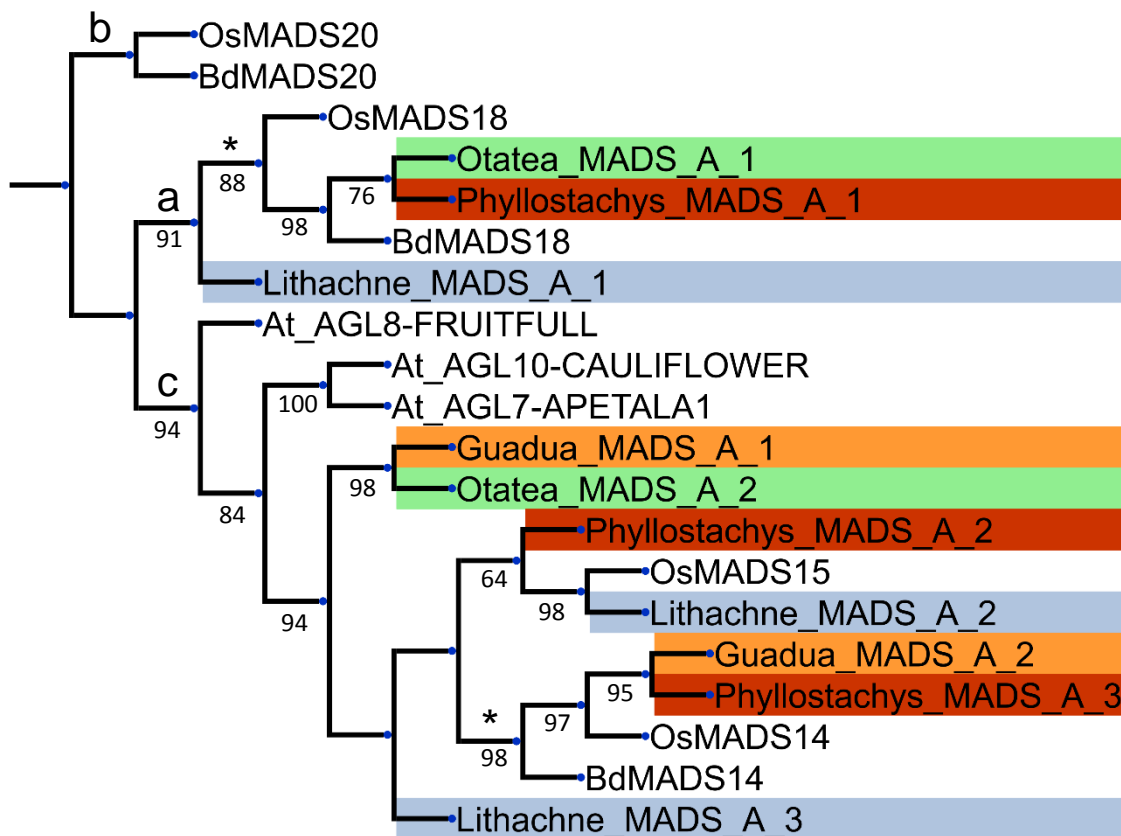

C/D

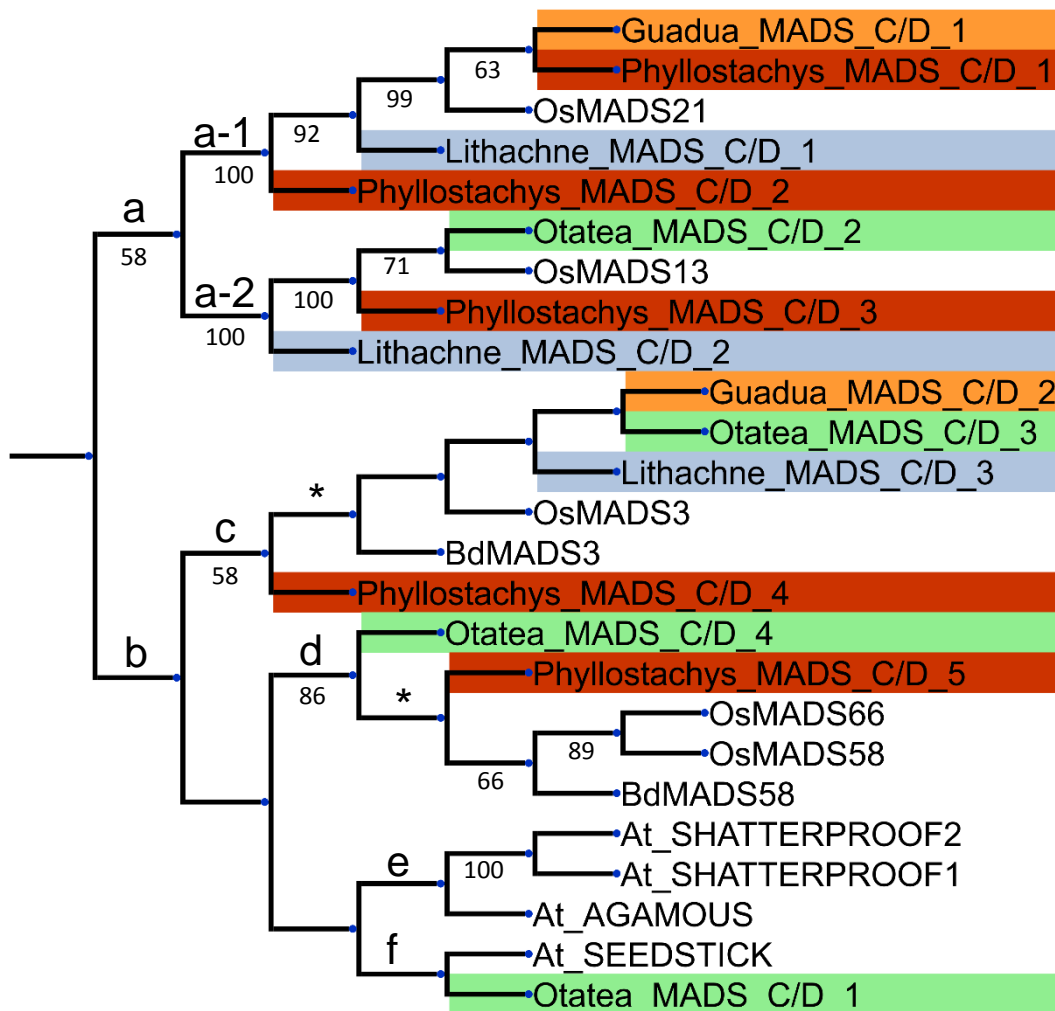

Supplement: Additional file 2: Figure S1: — Neighbor-joining gene tree representing the A and C/D-class MADS box genes. Gene copies assembled in this study are labeled by genus, colored according to taxa (orange: G. inermis, green: O. acuminata, dark red: P. aurea, blue: L. pauciflora) and numbered redundantly to distinguish copies. Reference gene copies are not colored, are abbreviated by binomial (At: Arabidopsis thaliana, Bd: Brachypodium distachyon, Os: Oryza sativa) and are numbered according to their labeling in Genbank. Nodes that were supported at over 50 % bootstrap are indicated. (PDF 331 kb) [file 12864_2016_2707_MOESM2_ESM.pdf]

# SOC

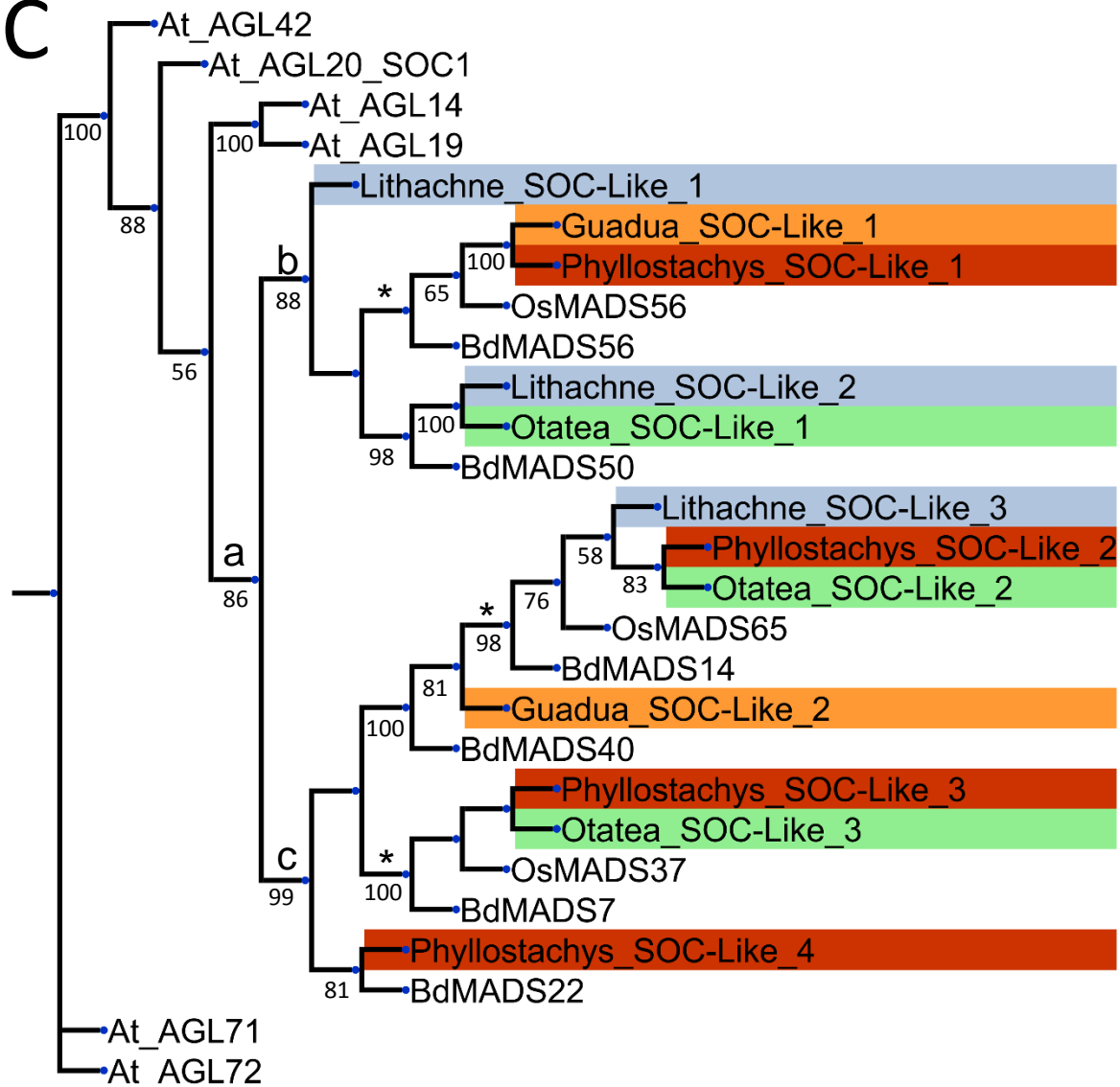

# E

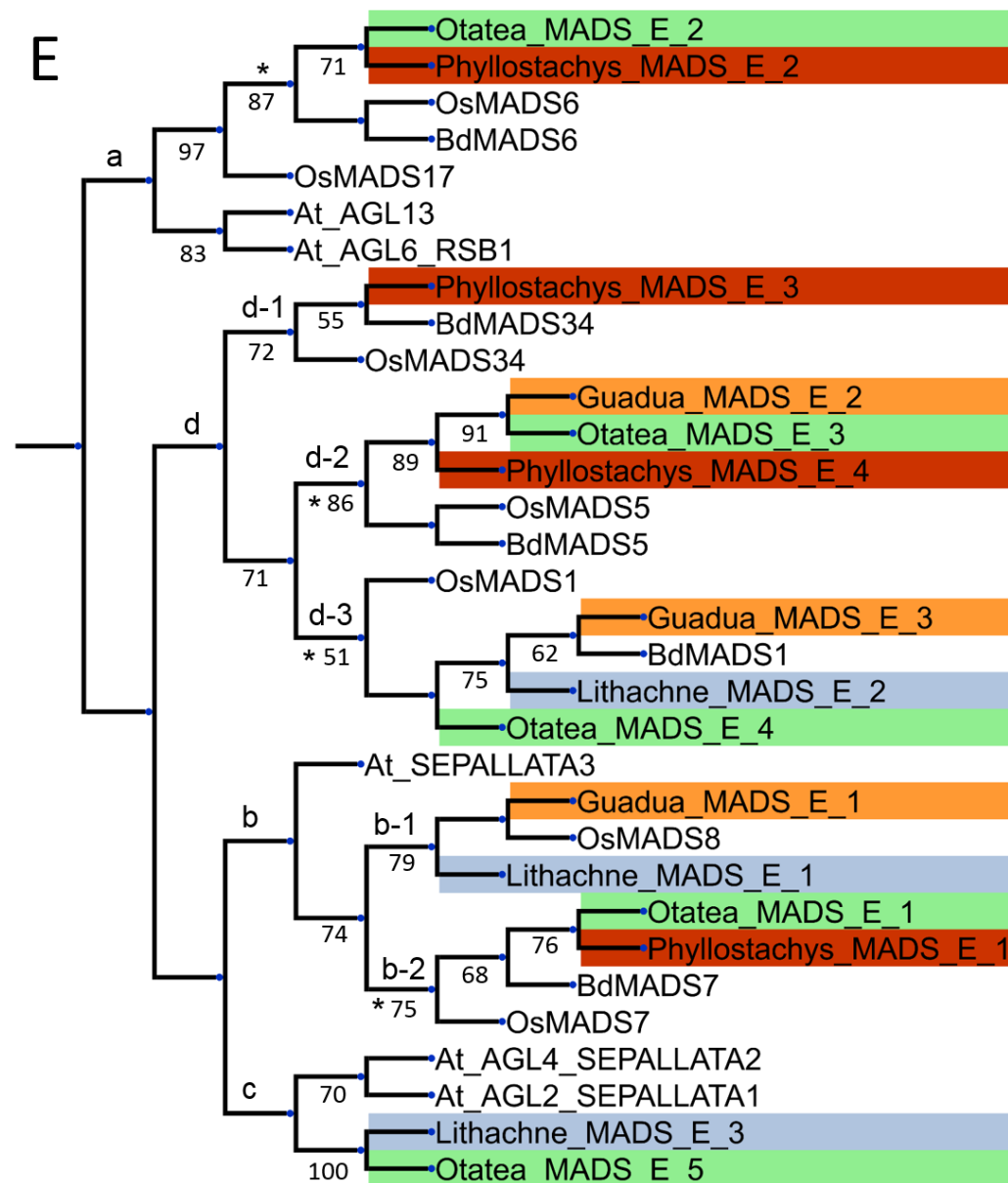

Supplement: Additional file 3: Figure S2: — Neighbor-joining gene tree representing the SOC-like and E-class MADS box genes. Gene copies assembled in this study are labeled by genus, colored according to taxa (orange: G. inermis, green: O. acuminata, dark red: P. aurea, blue: L. pauciflora) and numbered redundantly to distinguish copies. Reference gene copies are not colored, are abbreviated by binomial (At: Arabidopsis thaliana, Bd: Brachypodium distachyon, Os: Oryza sativa) and are numbered according to their labeling in Genbank. Nodes that were supported at over 50 % bootstrap are indicated. (PDF 465 kb) [file 12864_2016_2707_MOESM3_ESM.pdf]
